# Emergency surgery for fibroid expulsion complicated by pelvic organ prolapse: a case report

**DOI:** 10.1093/jscr/rjaf215

**Published:** 2025-04-14

**Authors:** Hirofumi Kawahara, Riho Yumisashi, Yuka Fukunishi, Masaki Kamio

**Affiliations:** Department of Gynecology, NHO Kagoshima Medical Center, 8-1, Shiroyama, Kagoshima-shi, Kagoshima-ken 892-0853, Japan; Department of Gynecology, NHO Kagoshima Medical Center, 8-1, Shiroyama, Kagoshima-shi, Kagoshima-ken 892-0853, Japan; Department of Gynecology, NHO Kagoshima Medical Center, 8-1, Shiroyama, Kagoshima-shi, Kagoshima-ken 892-0853, Japan; Department of Gynecology, NHO Kagoshima Medical Center, 8-1, Shiroyama, Kagoshima-shi, Kagoshima-ken 892-0853, Japan

**Keywords:** abnormal uterine bleeding, fibroid expulsion, myoma fibroid, pedunculated submucous fibroid, pelvic organ prolapse

## Abstract

Uterine fibroids are common benign gynecological tumors. Submucosal fibroids, which protrude into the uterine cavity, may sometimes extend through the cervical canal as pedunculated masses, causing abnormal uterine bleeding (AUB). However, co-occurrence of fibroid expulsion with pelvic organ prolapse (POP) is rare. We report the case of a 52-year-old multiparous woman who was referred to our hospital for the treatment of fibroid expulsion and complete uterine prolapse. Hysterectomy and colporrhaphy were deemed necessary to alleviate these symptoms. Preoperative anemia was managed using oral Relugolix, a gonadotropin-releasing hormone (GnRH) antagonist, to reduce uterine bleeding. On the 33rd day of treatment, the patient presented with severe uncontrollable vaginal bleeding, necessitating emergency surgery. Abdominal and vaginal surgical approaches have been used for total hysterectomy, colporrhaphy and perineoplasty. This case highlights the necessity of surgical innovation in treating coexisting fibroid expulsion and POP and the potential risk of AUB during GnRH antagonist therapy.

## Introduction

Uterine fibroids and benign solid smooth muscle tumors are classified according to their location into subserosal, intramural, and submucosal types [1]. Submucosal fibroids comprise ⁓5% of cases [2]. They affect 25% of women over 35 years of age, with ⁓50% being symptomatic [3], with symptomatic fibroids often involving submucosal fibroids [4]. Sometimes submucosal fibroids descend into the vaginal canal as pedunculated fibroids, a phenomenon termed “fibroid expulsion.” The frequency of fibroid expulsion remains unclear; however it has been reported to occur 2.5% of patients undergoing surgery for uterine fibroids [2]. Fibroid expulsion coexisting with pelvic organ prolapse (POP) has rarely been reported. Herein, we present the case of a premenopausal woman with concurrent complete uterine prolapse and fibroid expulsion who required surgical intervention for abnormal uterine bleeding (AUB) associated with a gonadotropin-releasing hormone (GnRH) antagonist.

## Case presentation

**Figure 1:**
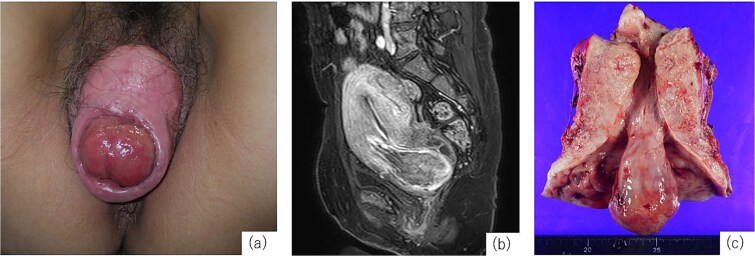
Examination findings. (a) Complete uterine prolapse with a mass protruding through the external cervical os. (b) Contrast-enhanced magnetic resonance imaging (T1-weighted) shows a submucosal fibroid originating from the anterior uterine wall descending through the cervical canal. (c) Resected specimen shows uterine cross-section showing a 3-cm stalked fibroid extending through the cervical canal.

A 52-year-old woman, gravida 4, para 3, with regular menstrual cycles presented with a 9-year history of vaginal discomfort and recent abnormal menstrual bleeding. She had a history of mild POP, but no prior treatment. Although the discomfort had not improved, she did not visit a gynecologist. She visited a gynecologist with complaints of increased menstrual bleeding and a drooping sensation and was diagnosed with fibroid expulsion and POP, and 2 months later, referred to our department. The gynecological assessment revealed fibroid expulsion with complete uterine prolapse, cystocele, rectocele and a prolapsed mass visible in the external cervical os (Fig. 1a). Contrast-enhanced magnetic resonance imaging (MRI) identified a pedunculated submucosal fibroid (69 × 42 × 50 mm) originating from the anterior uterine wall and extending through the cervical canal (Fig. 1b). Her blood tests showed anemia (hemoglobin [Hb]: 9.2 g/dL); therefore, preoperative management with relugolix (40 mg/day), a GnRH antagonist, was planned. On day 33 of treatment, the patient experienced heavy, uncontrolled vaginal bleeding and was admitted for emergency surgery. Given the significant size of the uterus and the fibroids, laparoscopic surgery and vaginal total hysterectomy (VTH) are deemed infeasible. Therefore, an abdominal approach was used. Abdominal total hysterectomy (ATH), bilateral salpingectomy, colporrhaphy, and perineoplasty. Surgery was performed with the patient in the lithotomy position. The uterus was markedly enlarged to the size of a large fist, and the uterovesical fold of the peritoneum was significantly descended owing to complete uterine prolapse, complicating its dissection and elevation. The uterine isthmus widened because of fibroid expulsion, obscuring the localization of the uterine vaginal junction. After treating the round, utero-ovarian, and cardinal ligaments, a vertical incision was made on the anterior vaginal wall to identify the distal margins of the vaginal vault. Colpotomy was then performed to excise the uterus. Subsequently, the procedure was transitioned to a vaginal approach to repair the bladder and rectal prolapse. The vaginal wall closure was completed after colporrhaphy. The operative time was 3 h and 57 min, with an estimated blood loss of 470 mL. The resected specimen revealed a pedunculated mass originating from the posterior uterine wall (Fig. 1c). On the fifth postoperative day, the indwelling urinary catheter was removed, and normal urination was confirmed. The patient was discharged on the 13th postoperative day. At the 1-month postoperative follow-up, the patient exhibited no abnormalities and was deemed to have recovered, concluding her treatment.

## Discussion

Fibroid expulsion occurs when a submucosal fibroid protrudes through the cervical canal, often becoming symptomatic, and is a common cause of AUB [1]. POP is the descent of the pelvic organs due to weakened support structures, influenced by genetic predisposition, neuropathy, myopathy, childbirth, and contributing factors such as obesity, smoking, pulmonary disease, and constipation [5], and is most prevalent among postmenopausal women [5]. The coexistence of fibroid expulsion and POP is rare, and only a few surgical case reports under different conditions have been published. Necrotic pedunculated prolapsed submucosal fibroids cases complicated by complete utero-vaginal prolapse treated with VTH are reported [6, 7]. Necrosis may occur due to compromised blood flow through the stalk; however, in our case, the stalk was 3 cm thick, which likely preserved the blood supply.

In the present, laparotomy, colporrhaphy, and perineoplasty were performed. The surgical options for fibroids and POP include vaginal and laparoscopic surgeries. However, in our case, fibroid expulsion confined within the cervical canal significantly widened the uterine isthmus, raising concerns of ureteral injury; therefore, VTH was deemed impractical. Thick stalk also poses a high risk of massive hemorrhage, making myomectomy and laparoscopic surgery infeasible in the emergency setting. Myomectomy in case of fibroid expulsion >5 cm can result in 30% conversion to hysterectomy due to uncontrolled bleeding, underscoring the risks associated with myomectomy [8]. Finally, ATH was selected. Because the fibroid obscured the location of the vaginal vault, a vertical incision was made on the anterior vaginal wall to identify the vaginal margins before colpotomy and uterine removal. The surgery also incorporated procedures for POP repair, thereby avoiding vaginal vault closure during the abdominal phase. Instead, colporrhaphy was performed to repair the cystocele and rectocele before completing vaginal vault closure.

GnRH analogs, including agonists and antagonists, are widely used in the management of infertility, uterine fibroids, and endometriosis. GnRH antagonists, such as relugolix, suppress estrogen and progesterone without inducing flare-up effects [9, 10]. Relugolix rapidly reduces fibroid and uterine size and demonstrates superior control of menstrual bleeding compared to the GnRH agonist leuprorelin [10]. However, its use for submucosal fibroids is associated with severe AUB. In our case, relugolix initially controlled the bleedings, but led to severe AUB on day 33 of treatment. Although effective in reducing the fibroid size and alleviating menorrhagia and pelvic pain, the risk of severe bleeding must be considered. Therefore, careful patient selection and awareness of these risks are critical when prescribing GnRH antagonists.

## Conclusion

This case highlights the importance of tailored surgical strategies for managing coexisting fibroid expulsion and POP. Surgeons should be vigilant about potential AUB during GnRH antagonist therapy and consider individual anatomical and pathological complexities when planning surgery.
